# Non-Coding RNAs in Cancer: An Interview with Dr. Martin Pichler

**DOI:** 10.3390/ijms17040605

**Published:** 2016-04-21

**Authors:** International Journal of Molecular Sciences Editorial Office

**Affiliations:** MDPI AG, Klybeckstrasse 64, CH-4057 Basel, Switzerland; ijms@mdpi.com

In this issue, we are pleased and honored to have an interview with Professor Martin Pichler, who is the Collection Editor for the *International Journal of Molecular Sciences* Topical Collection of “Regulation by Non-Coding RNAs” [1].


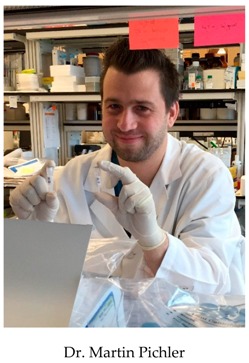


Professor Martin Pichler, MD, MSc, received his MD at the Medical University of Graz, and his Master degree in Molecular Biology at the Karl-Franzens University Graz, Austria. Dr. Pichler continued his cancer research training in the laboratory of Professor Gerald Hoefler and Professor Kurt Zatloukal, both Institute of Pathology, Medical University of Graz, Austria and in the lab of Professor George Adrian Calin at the University of Texas MD Anderson Cancer Center in Houston, USA. Finally, Dr. Pichler became a board-certified specialist in Internal Medicine, at the Division of Oncology, Medical University of Graz in Austria.

Currently, he is an Associate Professor at the Division of Oncology, Medical University of Graz, and the Head of the Research Unit for non-coding RNA and genome editing at the Medical University of Graz. He also holds an Adjunct Assistant Professor position at the University of Texas MD Anderson Cancer Center, Houston, Texas, USA.

Professor Pichler authored more than 135 peer-reviewed scientific articles and has received several awards and fellowships. The main focus in his own lab in Austria is the characterization of non-coding RNAs in cancer and their usefulness as novel diagnostic and prognostic biomarkers in cancer patients.

Here, in this interview, we are able to find out his opinions on the field of non-coding RNAs in cancer and discuss what publications Dr. Pichler would like to see in his Topical Collection.

**1.**  **How did you first become interested in non-coding RNAs and genome editing in cancer, and is there a particular person who has influenced you in your career?** 

The first time I realized the existence of long non-coding RNAs in cancer was in 2006, when I did my first postdoctoral fellowship in the lab of my previous boss (*i.e.*, Prof. Kurt Zatloukal from the Medical University of Graz, Austria) who characterized and discovered a novel transcript in liver cancer. This highly up-regulated in liver cancer (HULC) transcript was described by some of my lab mates at a time when the concept of long non-coding RNAs and how they influence cancer cells was quite new (see also Panzitt *et al.* [2]). Besides that, there were a lot of people interacting and influencing my career. Just to mention the most important ones, there was my supervisor at the undergraduate student level, a pathologist (*i.e.*, Professor Gerald Hoefler, Medical University of Graz), who taught me how to think scientifically, how to apply for grants and who remained a great mentor for many years , even after finishing my MD. Second, Professor George Adrian Calin (MD Anderson Cancer Center, Houston, Texas, USA) gave me a very specialized education in the field of non-coding RNAs. George Calin made several big discoveries in the field, including the first link between microRNAs and cancer, the first link between ultra-conserved genes and cancer, and the description of CCAT2, a long non-coding RNA in colorectal cancer. I succeeded a three years training in his lab and this scientific setting was one of the most impressive and informative trainings I have ever experienced. Two other people, Tony Gutschner and Hui Ling, both postdoctoral fellows at that time and very bright guys, were great colleagues and scientists who helped shape my expertise in the field of non-coding RNAs.

**2.**  **In your opinion, what challenges and developments can we expect to see in the next few years in the field of non-coding RNAs (for example, microRNAs and long non-coding RNAs) in human cancer?** 

Well, I think we have to separate these two categories of non-coding RNAs. First, let’s talk about microRNAs. These molecules were discovered 15 years ago in human cancer and several thousands of original scientific reports have been published so far. However, what this field really needs is a clinical application. There are three main areas for clinical application: Diagnosis, prognosis and therapeutics. For diagnosis, microRNAs have some interesting features such as chemical stability in body fluids or after chemical preservation in formalin, relatively easily applicable and cheap technical quantification methods, *etc.* However, there are some limitations including the lack of specificity and sensitivity, the background noise in biological samples, *etc.* So in my personal opinion, I do not foresee a dramatic improvement of routine clinical diagnosis or prognosis of any type of cancer by the use of microRNA-based assays in the next 5 to 10 years (but I hope I am wrong here…). Regarding the therapeutic field, though not proven yet, this seems to hold greater promise. The first clinical trials (not only in cancer) are underway and these first in human trials will probably give some basal information (toxicities and first signals for efficacy). However, there are several unsolved problems similar to other RNA-based therapeutic fields, including inefficient delivery systems, off-target effects (which might be very strong with microRNA-based drugs as they target several RNA targets in different organ systems) and the chemical backbone. For the long non-coding RNA field, besides the exciting discovery and characterization of molecular biology and mechanisms involved, the greatest potential is their usefulness as diagnostic cancer biomarkers. The PCA3 prostate cancer detection test is one first example of this.

**3.**  **How can RNA research contribute to meeting these challenges?** 

Understanding the pathophysiology, the molecular biology, the interaction of RNAs with DNA, other classes of RNAs and proteins and all the other efforts in the RNA research field will contribute to meeting the goals mentioned above.

**4.**  **As an experienced researcher in this field, what do you think is the most important aspect to consider when dealing with RNA research, bioinformatic target prediction and genome editing?** 

Saying this sentence, you have already summarized very important aspects: Current RNA research needs bright bioinformatics and genome editing techniques like CRISPR/Cas9. Especially CRISPR/Cas9 has already and will probably influence the RNA research field (but even more so in other areas) in an unpredictable dimension. The genome editing field is rapidly moving forward and there is currently no *Science*, *Cell* or *Nature* issue without a paper showing novel development, optimizations or pre-clinical applications for this technology. CRISPR/Cas9 is a technology that will bring the whole field of molecular biology and maybe medicine to the next level.

**5.**  **How can non-coding RNAs and genome editing be applied to other fields?** 

Well, there are similar approaches as mentioned above for non-coding RNAs in other fields especially in medicine. Just one example is a successful clinical phase II trial published in New England Journal of Medicine about the treatment of hepatitis C liver infection by a single micoRNA-based drug. Other examples include metabolic disorders including even orphan diseases. As mentioned above, again the therapeutic field is the one with the greatest hope and potential for clinical application in neuroscience, cardiovascular or metabolic diseases.

**6.**  **You are supervising a Topical Collection “Regulation by Non-Coding RNAs” for our journal. Are there any particular papers you would like to see in your Topical Collection?** 

Our topical collection comprises already more than 30 high quality manuscripts including original papers and review articles. I like to see reports from young scientists as well as established researchers reporting their new findings, presenting new concepts, summarizing current knowledge in a logical way and touching new areas in the field of non-coding RNAs. Cancer articles are welcome, but there is also interest in all other fields of medicine with a special focus on non-coding RNAs and genome editing techniques.

**7.**  **Do you have any suggestions or recommendations for young scientists, for instance, your students and young collaborators?** 

There are several aspects for young up-coming researchers they should consider.

First, select a mentor (PhD supervisor, postdoc position) and a lab that matches with your own main research interests and topic. Second, check if this lab and the mentor is productive (check the senior authorships and how many people of the lab are first author in the last three years and divide this by the number of people in the lab). Third, try to get in contact with lab people there and ask them about the place/mentor, *etc.* and see if this matches the way you want to work. Fourth, try to get as many opportunities as possible (congress presentations, awards, small first grants, and publications) in early career stage. Fifth, try to talk and communicate with your own lab colleagues and people from other labs. These contacts can be very helpful for future collaborations and acquiring different expertise. Sixth, visit many talks and scientific presentations (academics as well as industry), read every week the new titles of articles published in the big journals in your field as this is the only way you can get new information, up-dated knowledge and also a cross link to new developments, trends and other topics. Seventh, be happy and try to live a balanced life, you will only live once on this planet!
